# Relação entre Complacência Pulmonar Estática e Extubação após Cirurgia Cardíaca

**DOI:** 10.36660/abc.20240125

**Published:** 2024-04-15

**Authors:** Henrique Murad

**Affiliations:** 1 Universidade Federal do Rio de Janeiro Rio de Janeiro RJ Brasil Universidade Federal do Rio de Janeiro, Rio de Janeiro, RJ – Brasil

**Keywords:** Procedimentos Cirúrgicos Cardíacos/métodos, Extubação/métodos, Complacência Pulmonar, Insuficiência Respiratória, Cuidados Pós-Operatórios

O excelente trabalho "Relação entre Complacência Pulmonar Estática e a Falha de Extubação em Pacientes no Pós-Operatório de Cirurgia Cardíaca"^1^ publicado nos Arquivos Brasileiros de Cardiologia, nos dá mais uma ferramenta para auxiliar na decisão para permitir extubação com sucesso mantido, no pós-operatório de cirurgia cardíaca. Os autores estudaram 102 pacientes submetidos a diversas operações cardíacas com circulação extracorpórea e observaram necessidade de reintubação em 25 pacientes (24,51%) após 48 horas de extubação. Os pacientes que falharam na extubação tiveram complacência pulmonar mais baixa do que os que não tiveram necessidade de serem reintubados. O ponto de corte da complacência pulmonar estática para uma extubação sem necessidade de reintubação foi de 41 ml/ cm H_2_O, sendo o normal entre 60 e 100 ml/ cm H_2_O. Na análise de regressão logística múltipla a falha de extubação foi 9,1 vezes maior em pacientes com complacência pulmonar inferior a 41 ml/ cm H_2_O. Também observaram que a complacência pulmonar estática mais baixa foi acompanhada de pior troca gasosa. Recomendam que a medida da complacência pulmonar estática deva ser incluída na avaliação pós-operatória do paciente submetido à cirurgia cardíaca.

Parte do material deste artigo integrou a dissertação de mestrado da Dra. Thais da Silva Brito, realizada sob a orientação da Professora Doutora Desanka Dragosavac, na Universidade Estadual de Campinas.^2^

Necessidade de nova intubação pós extubação é extremamente danosa e ocasiona maior tempo de UTI, maior tempo em respirador, maior tempo de internação e maior mortalidade. A menor complacência pulmonar estática e reintubação são demonstrações de piora clínica e respiratória. Evitar extubação precoce é importante para não acrescentar problemas a um paciente em pós-operatório de cirurgia cardíaca.^2^

Este trabalho merece alguns comentários: 1 - Com um número maior de pacientes sendo extubados na sala de operações, as medidas de complacência pulmonar estática, fora do ambiente de pesquisa, deveriam provavelmente começar a serem tomadas no centro cirúrgico.^3^ 2 - O problema não é a reintubação em si, mas sim a etiologia do problema que levou à falha de extubação. Seria interessante analisar o comportamento tardio da complacência pulmonar estática e conhecer os motivos que levaram à falha de extubação.

Atualmente muito se tem publicado sobre programas de melhor recuperação após cirurgia cardíaca ( *ERAS- Enhanced recovery after surgery* ) e a extubação precoce faz parte destes estudos.^4^ O importante, no entanto, não é a rapidez da extubação, mas a adequação para fazê-lo no momento certo.^5^ Neste contexto a medida da complacência pulmonar estática pode ser de grande valor na tomada de decisão a favor da extubação precoce.
