# Supplementary material for: Relação entre Complacência Pulmonar Estática e Extubação após Cirurgia Cardíaca
Source: Arq Bras Cardiol. 2024 Apr 15;121(2):e20240125. [Article in Portuguese] doi: 10.36660/abc.20240125 (PMC11081084; doi:10.36660/abc.20240125)
Supplement: Translation

# Translation: Relationship between Static Lung Compliance and Extubation After Cardiac Surgery

**Authors:** Henrique Murad

**Affiliations:** 1 Rio de Janeiro RJ Brasil Universidade Federal do Rio de Janeiro, Rio de Janeiro, RJ – Brasil

**Keywords:** Cardiac Surgical Procedures/methods, Extubation/methods, Lung Compliance, Respirator Insuficiency, Post Operative Care

The excellent paper "Relationship between static lung compliance and extubation failure in postoperative cardiac surgery patients"^1^published in the *Arquivos Brasileiros de Cardiologia* gave one additional tool to make more precise decisions for successful and maintained extubation in postoperative cardiac surgery.

The authors have studied 102 patients submitted to several types of cardiac surgery under extracorporeal circulation and have found the need for reintubation in 25 patients (24,5%) after the first 48 hours of primary extubation. Extubation failure patients have had lower static lung compliance than non-failure patients. The cut-off point for the reintubation was a static lung compliance of 41 mi/cm H_2_O or lower, with a normal range between 60 and 100 ml/cm H_2_O. Using multiple regression analysis they had observed that extubation failure was 9,1 times greater in patients with static lung compliance equal to or less than 41 ml/cm H_2_O. They have also observed that lower static lung pulmonary compliance patients have worse oxygenation. They recommend that static lung compliance should be included in the postoperative evaluation of cardiac surgery patients, before extubation. This paper is part of the MSc thesis of Dr. Thais da Silva Bento, under the orientation of Dr. Desanka Dragosavac at the Universidade Estadual de Campinas.^2^

The need for new intubation in the postoperative cardiac surgery patient is extremely deleterious and may provoke more ICU time, more respirator time, greater admission time, and greater mortality. Lower static lung compliance and reintubation are demonstrations of clinical and respiratory compromise. To avoid too early extubation is mandatory to avoid problems to a postoperative cardiac surgical patient.^2^

Some remarks could be made on this paper: 1 - There is nowadays an increasing number of patients being extubated in the operating room at the end of the surgical procedure. Static lung compliance should probably be measured in the operating room at the end of the operation, before any attempt of extubation.^3^2 - The problem is not the reintubation by itself, but the reason why the reintubation was needed. It would be interesting to know the reasons for extubation failure and what was the late static lung compliance behavior.

In recent times much has been published on enhancing recovery after surgery (ERAS) and early extubation is part of every ERAS protocol.^4^The important is not the rush for extubation, but the readiness to do so.^5^Static lung compliance measurement could be a valuable help in the early extubation decision.
